# Should a Sentinel Node Biopsy Be Performed in Patients with High-Risk Breast Cancer?

**DOI:** 10.4061/2011/973245

**Published:** 2011-08-10

**Authors:** Kenneth D. Westover, M. Brandon Westover, Eric P. Winer, Andrea L. Richardson, J. Dirk Iglehart, Rinaa S. Punglia

**Affiliations:** ^1^Harvard Radiation Oncology Program, Boston, MA 02115, USA; ^2^Department of Neurology, Dana-Farber Cancer Institute, Brigham and Women's Hospital and Harvard Medical School, Boston, MA 02115, USA; ^3^Department of Medical Oncology, Dana-Farber Cancer Institute, Brigham and Women's Hospital and Harvard Medical School, Boston, MA 02115, USA; ^4^Department of Pathology, Dana-Farber Cancer Institute, Brigham and Women's Hospital and Harvard Medical School, Boston, MA 02115, USA; ^5^Department of Surgery, Dana-Farber Cancer Institute, Brigham and Women's Hospital and Harvard Medical School, Boston, MA 02115, USA; ^6^Department of Radiation Oncology, Dana-Farber Cancer Institute, Brigham and Women's Hospital and Harvard Medical School, Boston, MA 02115, USA

## Abstract

A negative sentinel lymph node (SLN) biopsy spares many breast cancer patients the complications associated with lymph node irradiation or additional surgery. However, patients at high risk for nodal involvement based on clinical characteristics may remain at unacceptably high risk of axillary disease even after a negative SLN biopsy result. A Bayesian nomogram was designed to combine the probability of axillary disease prior to nodal biopsy with customized test characteristics for an SLN biopsy and provides the probability of axillary disease despite a negative SLN biopsy. Users may individualize the sensitivity of an SLN biopsy based on factors known to modify the sensitivity of the procedure. This tool may be useful in identifying patients who should have expanded upfront exploration of the axilla or comprehensive axillary irradiation.

## 1. Introduction

In breast cancer, metastases to the axilla are associated with an increased risk of distant micrometastatic disease [1–3]. Sentinel lymph node (SLN) biopsy has become standard practice for evaluating the axilla in patients without palpable lymph nodes [4]. This procedure involves injection of a tracer, usually a radioactive colloid, alone or in combination with dye, into the tissue surrounding a tumor. Lymph nodes with evidence of uptake are surgically removed. The SLN procedure typically yields 1–5 nodes for pathologic examination whereas full axillary lymph node dissection (ALND) can yield greater than 20 nodes when taken to completion. On the other hand, SLN biopsy is associated with less pain, lower rates of postsurgical lymphedema, and better arm mobility when compared to full ALND [4]. Many patients with a positive SLN biopsy go on to have ALND for both diagnostic and therapeutic purposes. However, there is growing evidence that axillary irradiation may be used instead of ALND in select cases with excellent results [5]. Indeed a recent randomized trial showed that ALND offered no benefit over SNL biopsy in terms of local control or survival to women with early clinical stage breast cancer who also received radiation therapy [6]. 

Like any diagnostic test, SLN biopsy can yield false-negative results. Several factors can affect the sensitivity of an axillary SLN biopsy (Table 1). Large tumors have been associated with decreased sensitivity (equivalently, higher false-negative rates), perhaps because they access a greater number of local lymphatic pathways and therefore have the potential for spreading to a larger distribution of nodes [7, 9]. Age-related fatty changes in nodes may decrease the capacity for dye or isotope uptake [8]. Medially located tumors may drain more frequently to internal mammary nodes than tumors located centrally or in the lateral breast [8]. Finally, two prospective studies suggest that the sensitivity of SLN biopsy correlates with the number of nodes removed [10, 12]. It should be noted that as techniques and protocols have improved, the sensitivity of SLN biopsy has generally improved. Nevertheless, the procedure remains imperfect, and most recent studies demonstrate false-negative rates in the range of 5–10% for small tumors [10, 12]. 

Breast cancer risk calculators are being increasingly used to guide adjuvant systemic and local treatment [14–17]. Using such a calculator, the probability of axillary nodal involvement for a given patient can be estimated prior to SLN biopsy based on a number of prognostic factors including age, tumor size, and histopathological features of the breast cancer. One example of such a risk calculator was developed at Memorial Sloan Kettering (MSKCC) and is available for use online. This calculator was originally intended to spare *low-risk* patients an SLN biopsy when the probability of nodal involvement is low [14]. However, this calculated probability may also benefit *high-risk* patients when used in combination with estimates of the false-negative rate of SLN biopsy to calculate the risk of having residual nodal disease in the setting of a negative SLN. We developed a nomogram that combines this probability of axillary disease with estimates of the sensitivity of SLN biopsy, to calculate the risk of residual axillary disease despite a negative SLN biopsy.

## 2. Methods

Bayes' rule combines the pretest probability of a given diagnosis with results from a test with known sensitivity and specificity to yield a posttest probability of having the diagnosis. In this analysis, the pretest probability is the probability of having axillary disease prior to any nodal evaluation; the posttest probability is the probability of axillary disease given a negative SLN biopsy; the false-negative rate (1-sensitivity) of SLN biopsy can be estimated from Table 1. The specificity of SLN biopsy is by definition equal to one (equivalently, the probability of a positive SLN in the absence of lymph node involvement disease is zero).

In this setting, Bayes' rule takes the following form:

(1) $$\text{Post}=\text{pre}*\frac{\left(1-\text{sens}\right)}{\text{pre}*\left(1-\text{sens}\right)+\left(1-\text{pre}\right)\left(\text{spec}\right)}.$$

With “post” and “pre” defined as posttest and pretest probabilities, respectively, “sens” defined as the sensitivity of SLN biopsy, and “spec” defined as the specificity of the procedure, which in our situation is 1.

Using this formula we can estimate the probability that a breast cancer patient has residual axillary disease despite a negative SLN biopsy. A Bayesian nomogram was constructed in MATLAB (MathWorks, v7.8) using the mathematical relationship above. We used a range of pretest probabilities from 5 to 85% and a 4 estimates of sensitivity (80%, 85%, 90%, and 95%) for the SLN biopsy procedure.

## 3. Results

**Figure 1:**
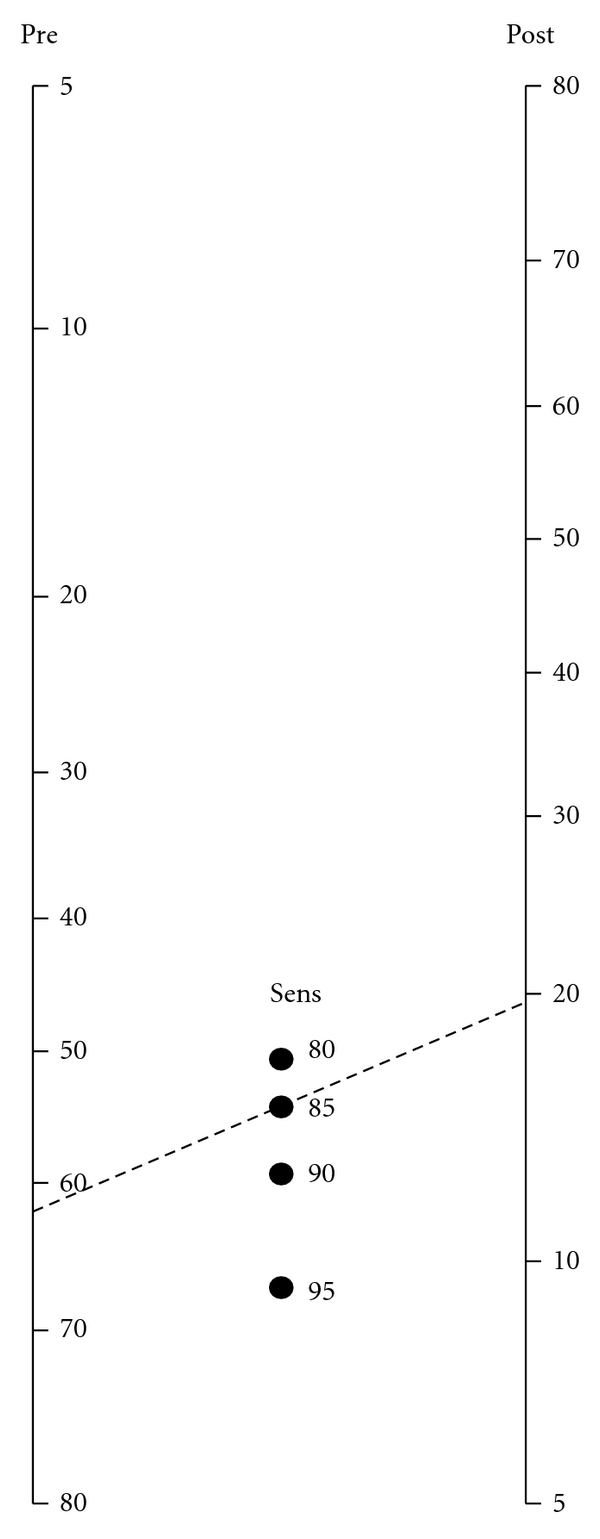
Bayesian nomogram for probability of metastatic disease as a function of pretest probability and negative SLN biopsy. The “pre” or pretest probability of axillary disease can be estimated using a risk calculator such as the one described [14]. The “sens” or sensitivity of SLN biopsy can be estimated using Table 1. Positions for the central dots are calculated assuming a SLN biopsy sensitivity of 80%, 85%, 90%, or 95%; specificity is assumed to be 100%. The calculation for the example patient is shown by the dotted line: if we assume sensitivity of 85% for SLN biopsy and a pretest probability of 62%, the posttest probability for axillary disease is 20% for this patient even in the presence of a negative SLN biopsy.

We created a Bayesian nomogram for the probability of axillary nodal involvement despite a negative SLN biopsy (Figure 1). The nomogram was designed to be flexible in order to accommodate a variety of clinical scenarios. A range of sensitivity values are displayed along the middle axis as discrete points; the appropriate value for a given patient can be estimated using Table 1. A line drawn through a given pretest probability and sensitivity point will intersect with the appropriate posttest probability (probability of having residual axillary disease despite a negative SLN biopsy) on the right-hand axis. 

For example, the nomogram can be used to calculate the risk of residual nodal disease in a 62-year-old woman who presents after a lumpectomy revealing a 1.5 cm, grade 2 invasive ductal carcinoma, hormone receptor-negative, with no lymphovascular invasion and an SLN biopsy yielding 3 negative nodes. According to the MSKCC model her risk of axillary disease prior to SLN biopsy is 19%. Assuming 95% for the sensitivity of SLN biopsy in this situation, the nomogram reveals that the probability of having residual axillary disease is 1.2%. Even if the sensitivity of SLN biopsy was assumed to be 85%, the posttest probability remains low at 3.4%.

Likewise, the nomogram can be used to calculate the probability of residual axillary disease despite a negative SLN biopsy in a woman at higher risk of axillary involvement. A 64-year-old patient with a 2 cm, grade 3, hormone receptor-positive, invasive ductal carcinoma with lymphovascular invasion has a 62% pretest probability of metastases to the axilla based on her pathology. Using a sensitivity of 85% for SLN biopsy, the nomogram reveals that the probability of residual axillary nodal involvement is 20%. In other words, despite a negative SLN biopsy, she still has a 20% probability of finding metastatic disease with completion ALND. If the SLN biopsy procedure has a 95% sensitivity the nomogram reveals that the risk of additional axillary disease after a negative SLN biopsy decreases to 7.5%.

Table 2 provides a summary of findings for the posttest probability of residual axillary disease despite negative SLN biopsy for a range of pretest probabilities of axillary disease prior to SLN biopsy at each of 3 different sensitivities for the SLN procedure. 

## 4. Discussion

The presence of axillary disease is the most important prognostic factor in breast cancer. Disease in the axilla can indicate biological aggressiveness and extent of tumor involvement, often suggesting systemic spread and the need for additional therapy. In addition, the link between locoregional disease control and overall survival in breast cancer has been firmly established by meta-analyses of randomized data [18]. The potential importance of ALND in select patients is underscored by a recent analysis which suggested a survival benefit for women with macroscopic nodal disease that received ALND as compared to women with SLN biopsy alone [19]. Additionally the NCIC CTG MA.20 trial which randomized patients with high-risk breast cancer to postoperative whole-breast (WB) radiotherapy alone versus WB plus regional nodal irradiation showed improved locoregional control and an even greater improvement in distant disease control in the arm with regional nodal irradiation [20]. Therefore, the risks of more extensive axillary treatment versus the risks of missing occult disease must be carefully considered.

Our nomogram is intended to be flexible and enable increased personalization of cancer care. Specifically, our analysis is most applicable to two clinical scenarios and argues that

1. for a patient who had a negative SLN biopsy, but still has a high posttest probability of axillary disease, comprehensive axillary radiation may be warranted;
2. for a patient who has not yet undergone any axillary surgery, who has a high pretest probability of positive axillary nodes based on clinical features and who also has clinical characteristics that might decrease the sensitivity of SLN biopsy (as in Table 1), expanded axillary assessment up front may be warranted.

These conclusions seek to limit overtreatment in the form of multiple surgeries (SLN biopsy followed by ALND) and undertreatment in the form of omission of axillary radiation in breast patients with high-risk disease. 

We created a nomogram to estimate the risk of residual axillary disease despite a negative SLN biopsy as a function of the sensitivity of the SLN biopsy procedure and the pretest probability of axillary disease prior to axillary evaluation. Our nomogram reveals that for patients with a high pretest probability of axillary metastases and factors associated with a lower SLN biopsy sensitivity, the posttest probability of axillary disease often remains high despite a negative biopsy. While SLN biopsy is the appropriate test for most breast cancer patients, a preemptive expanded assessment of the axilla may be a better choice for high-risk patients or in cases where an SLN biopsy is predicted to be less sensitive (Table 1). 

In contrast to other probability calculators which estimate the risk of nonsentinel axillary nodal disease in a woman with breast cancer only *after* a positive SLN, our decision tool estimates the probability of nonsentinel axillary nodal disease *without* prior pathologic assessment of the axilla [15–17, 21]. The posttest probability obtained from the nomogram presented can then be assessed to be acceptable or not based on the specific clinical scenario. For example, a predicted posttest probability greater than 20–25% may warrant consideration of a more thorough axillary assessment upfront. Similarly, in cases where a negative SLN biopsy has already been obtained, a posttest probability of greater than 10% may suggest the need for the addition of axillary radiation. 

In patients identified by the nomogram to have an unacceptably high-risk of residual axillary disease despite negative SLN biopsy, more aggressive preemptive exploration of the axilla will necessarily mean a higher risk of lymphedema, nerve injury and general surgical complications compared to SLN biopsy; nevertheless, with modern ALND where only levels I-II are removed, these risks are less than observed historically [20]. Another option may be a less morbid lymph node sampling procedure as was reported in the UK, where at least 4 palpable lymph nodes are obtained by dissection starting at the axillary tail [22]. For patients where a negative SLN biopsy has already been obtained, but a high posttest probability of axillary disease remains, the addition of axillary radiation therapy could be considered in lieu of completion axillary dissection.

Use of this nomogram after a breast biopsy is not expected to result in excessive axillary treatment because it is likely to slightly underestimate rather than overestimate the probability of residual nodal disease. This is because the MSKCC risk calculator used above to obtain a pretest probability of having nodal disease was validated with information from complete pathologic specimens whereas, in practice, physicians are likely to substitute incomplete biopsy specimens such as findings from core needle biopsy, resulting in underdetection (due to undersampling) of certain negative prognostic factors, such as lymphovascular invasion, multifocality, and higher-grade tumor areas, which if found, increase the probability of having nodal disease. Therefore, use of only biopsy information would lead to underestimation of the pretest probability of having axillary involvement, which in turn would lead to underestimation of the posttest probability when using the nomogram. Another possible source of error associated with the use of incomplete biopsy specimens is that tumor size estimate entered into the MSKCC risk calculator must be estimated indirectly, based on imaging. However, assessment of tumor size by MRI, mammography and ultrasound does appear to correlate well with size as determined by pathologic exam [23, 24]. 

Although the recently published ACOSOG Z0011 and NSABP B-32 trials show similar rates of local control in both their SLN alone and ALND arms, the patients included in these trials were conservatively chosen and by definition had a low-risk profile [6, 25]. For the average patient in these trials, the risk of additional nodal disease after a negative SLN biopsy according to our nomogram would be under 6% even when using a low value of 85% as the sensitivity for SLN biopsy. Therefore our nomogram would not change the management of the average patient on these trials. 

For high-risk patients systemic therapy recommendations are also unlikely to change based on the output of this nomogram, because many of the factors which prompt addition of adjuvant therapy are the same factors that increase the pretest (and therefore also posttest) probability of having axillary disease. However, with regard to local management of the axilla, using this nomogram may change management, especially in light of the NCIC CTG MA.20 trial which included high-risk patients and suggests that residual untreated nodal disease may affect distant disease even in the absence of a clinically evident nodal recurrence. 

This nomogram is anticipated to apply to the small proportion of breast cancer patients who present with clinically high-risk disease. Randomized studies for this subset are difficult to perform because most breast cancer patients present with early-stage disease. Therefore, Bayesian estimation of risk based on a mathematically sound extrapolation from available data is especially useful in this clinical situation. Our nomogram is a particularly important tool for this group of high-risk patients because they may benefit from more extensive surgery or axillary radiation.

## Figures and Tables

**Table 1:** Factors influencing the sensitivity of SLN biopsy.

Factor	Sensitivity	Reference
T1	89.7–93.3%	[4, 7, 8]
T2-T3	82.0–92.6%	[7, 9]
Grade		[10]
1	95.7%	
3	90.4%	
Skill of surgeon	72.4–100%	[8]
Method		[11]
Combined dye and isotope	86.3–96.0%	
Dye	85.7–90.4%	
Isotope	86.3–97.8%	
Number of SLN removed		[10, 12]
1	82.3–89.1%	
3	93.1–98.9%	
5	99%	
Medial tumor	Decreases	[8]
Age > 50	Decreases	[8]
Obesity	Decreases	[10, 13]

**Table 2:** Calculated posttest probability of residual axillary disease despite negative SLN biopsy for a range of pretest probabilities for axillary disease with varying sensitivities of SLN biopsy.

	Posttest probability of axillary disease		
Pretest	Sens 0.85	Sens 0.90	Sens 0.95
0.05	0.008	0.005	0.003
0.1	0.016	0.011	0.006
0.2	0.036	0.024	0.012
0.3	0.060	0.041	0.021
0.4	0.091	0.063	0.032
0.5	0.130	0.091	0.048
0.6	0.184	0.130	0.070
0.7	0.259	0.189	0.104
0.8	0.375	0.286	0.167
0.9	0.574	0.474	0.310
